# Supplementary material for: Comparative transcriptome analysis of emerging young and mature leaves of Bienertia sinuspersici, a single-cell C4 plant
Source: PeerJ. 2025 Apr 29;13:e19282. doi: 10.7717/peerj.19282 (PMC12047216; doi:10.7717/peerj.19282)
Supplement: Supplemental Information 1 — Emerging Young (EY) and Mature (M) Bienertia sinuspersici leaf materials that were used in this study. Scale bar = 1 cm. Adapted from Figure 1B: Offermann S, Friso G, Doroshenk KA, Sun Q, Sharpe RM, Okita TW, Wimmer D, Edwards GE, van Wijk KJ. Developmental and Subcellular Organization of Single-Cell C. Photosynthesis in Bienertia sinuspersici Determined by Large-Scale Proteomics and cDNA Assembly from 454 DNA Sequencing. J Proteome Res. 2015 May 1;14(5):2090-108. doi: 10.1021/pr5011907. Epub 2015 Apr 9. PMID: 25772754. [file peerj-13-19282-s001.pptx]

## Slide 1
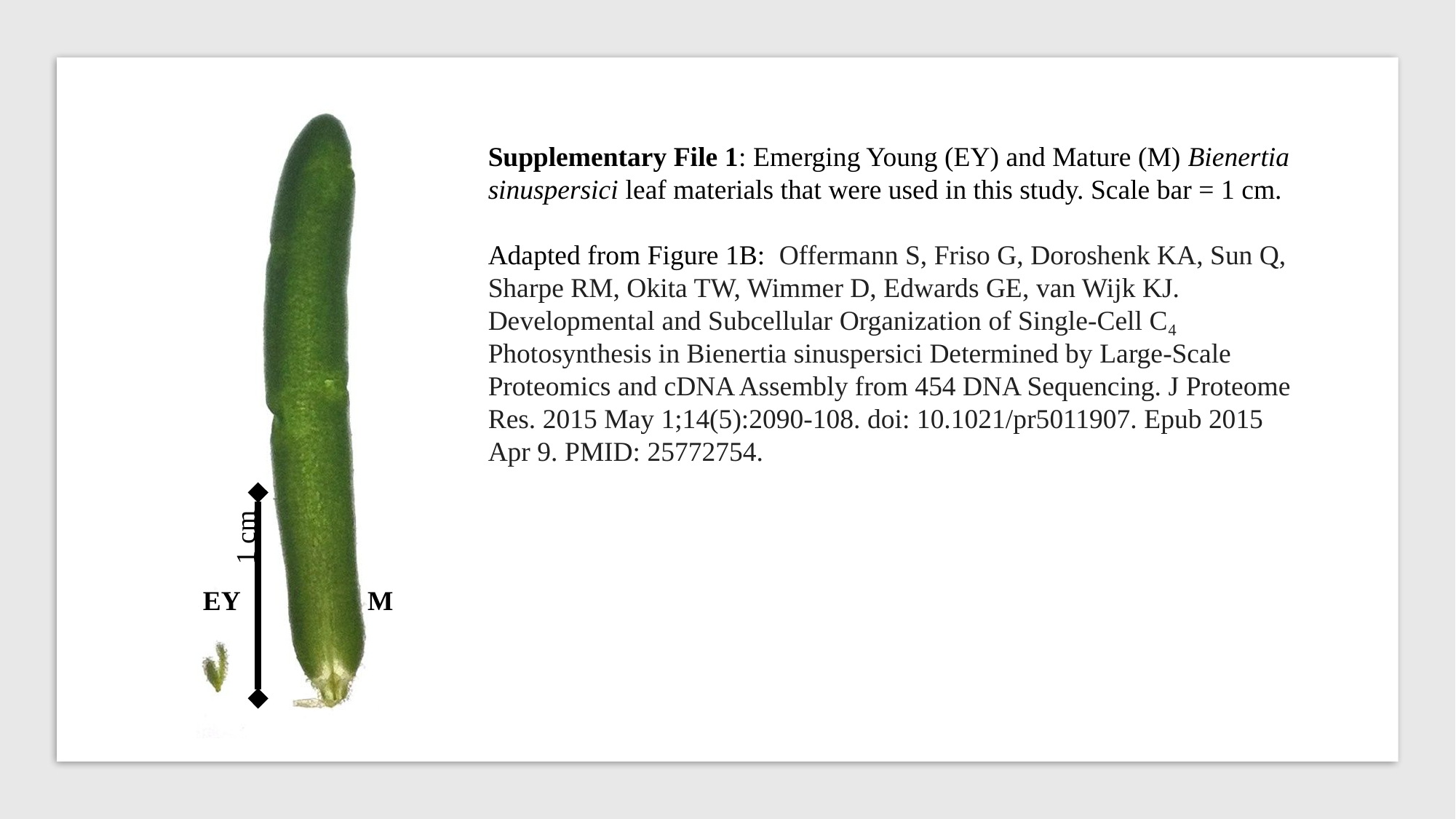

1 cm
EY
M
Supplementary File 1: Emerging Young (EY) and Mature (M) Bienertia sinuspersici leaf materials that were used in this study. Scale bar = 1 cm.
Adapted from Figure 1B: Offermann S, Friso G, Doroshenk KA, Sun Q, Sharpe RM, Okita TW, Wimmer D, Edwards GE, van Wijk KJ. Developmental and Subcellular Organization of Single-Cell C₄ Photosynthesis in Bienertia sinuspersici Determined by Large-Scale Proteomics and cDNA Assembly from 454 DNA Sequencing. J Proteome Res. 2015 May 1;14(5):2090-108. doi: 10.1021/pr5011907. Epub 2015 Apr 9. PMID: 25772754.
